# P-2159. Potential Application of a Clinical-Grade Next Generation Sequencing Assay for Detection of Bacterial Vaginosis Associated Organisms in Urine

**DOI:** 10.1093/ofid/ofae631.2313

**Published:** 2025-01-29

**Authors:** Mara Couto-Rodriguez, Sol Rey, Heather L Wells, Eduardo J Morfa Romero, Anna Plourde, Michael Augenbraun, Tiara Rivera, Xavier O Jirau Serrano, John C Papciak, Caitlin Otto, David C Danko, Christopher E Mason, Niamh B O’Hara, Dorottya Nagy-Szakal

**Affiliations:** Biotia Inc., Brooklyn, New York; Biotia Inc., Brooklyn, New York; Biotia, New York, New York; SUNY Downstate Health Sciences University, Brooklyn, New York; SUNY Downstate Medical Center, Brooklyn, New York; SUNY Downstate Medical University, Brooklyn, NY; Biotia, New York, New York; Biotia Inc., Brooklyn, New York; Biotia Inc., Brooklyn, New York; Biotia Inc., Brooklyn, New York; Biotia, New York, New York; Biotia Inc., Brooklyn, New York; Biotia Inc., Brooklyn, New York; Biotia Inc., Brooklyn, New York

## Abstract

**Background:**

Bacterial vaginosis (BV) is the most common cause of vaginal dysbiosis in women affecting about 21.2 million women ages 14-49 in the United States. Vaginal swabs are typically used for BV diagnosis using a combination of microscopy and PCR tests. Timely and precise diagnostics is essential for enhancing patient outcomes and minimizing antibiotic prescription. However, limitations persist within thediagnostic approaches currently employed in clinical practice. BIOTIA-ID Urine NGS Assay was assessed as a tool to diagnose BV from urine by developing an (NGS)-based Nugent score based on compositional dynamics of lactobacilli and BV-associated organisms.

**Methods:**

Healthy donor (HD, n=20) and culture negative (CN, < 100k CFU/mL) urine specimens (n=201) were collected from patients with pyuria and UTI symptoms and processed with the BIOTIA-ID Urine NGS Assay including DNA extraction, library preparation, Illumina-NextSeq sequencing and BIOTIA-DX analysis to identify urogenital pathogen infections. Findings were correlated with clinical metadata (Advarra Pro00038083, IRBNet 1950413-1).

**Results:**

BIOTIA-ID yielded an average of 2.15M microbial reads per sample with 100% passing laboratory QC and classified 62% of the CN-specimens as positive for dysbiosis/infection while HD specimens were negative. *Gardnerella vaginalis* (21.4%), *Prevotella* (14.9%), *and Aerococcus* species (6.9%) were the most often detected organisms in NGS-positive samples while 70% of HD were predominantly *Lactobacillus* species. 65% of the *Gardnerella* positive specimens were symptomatic women of reproductive age (18-49 age group) and 29.3% took a course of antibiotics 30 days prior to urine collection. Development and evaluation of an NGS-based Nugent score revealed significantly higher scores in the urine of BIOTIA-ID *Gardnerella* positive specimens (p< 0.0001).

**Conclusion:**

Similar to PCR studies, here we demonstrate that a urine specimen, which is noninvasive, and the incorporation of an NGS-Nugent score has potential application for BV diagnostics. Prospective clinical utilization studies in which collection of vaginal swabs and urine from both HD and clinically BV-positive patients are warranted to further develop and validate BIOTIA-ID as a BV-diagnostic assay.

**Disclosures:**

Mara Couto-Rodriguez, MS, Biotia: Employee|Biotia: Stocks/Bonds (Private Company)|Biotia Inc: Employee|Biotia Inc: Stocks/Bonds (Private Company) Sol Rey, BS, Biotia: Employee|Biotia: Stocks/Bonds (Private Company) Heather L. Wells, MPH, PhD Candidate, Biotia, Inc.: Employee Tiara Rivera, B.S., Biotia: Employee|Biotia: Stocks/Bonds (Private Company) Xavier O. Jirau Serrano, MS, Biotia Inc: Employee|Biotia Inc: Stocks/Bonds (Private Company) John C. Papciak, BS, Biotia: Employee|Biotia: Stocks/Bonds (Private Company) David C. Danko, Ph.D., Biotia Inc: Employee|Biotia Inc: Stocks/Bonds (Private Company) Christopher E. Mason, PhD, Biotia: Board Member|Biotia: Honoraria|Biotia: Ownership Interest|Biotia: Stocks/Bonds (Private Company) Niamh B. O'Hara, PhD, Biotia: Board Member|Biotia: Two patents related to genomic sequence analysis of microbial species|Biotia: Ownership Interest|Biotia: Stocks/Bonds (Private Company) Dorottya Nagy-Szakal, MD PhD, Biotia Inc: Employee|Biotia Inc: Stocks/Bonds (Private Company)

